# Understanding the Evidence‐to‐Practice Gap in Persistent Pain Management

**DOI:** 10.1155/prm/6171019

**Published:** 2026-10-07

**Authors:** Virginia K. Henderson, Christopher Covert, Ryan Pontiff, Rebecca Greenwood

**Affiliations:** ^1^ College of Nursing, Health and Human Services Texas A&M University-Texarkana Texarkana Texas USA tamut.edu; ^2^ Bert Bradford School of Health Sciences University of Charleston Charleston West Virginia USA ucwv.edu; ^3^ School of Health Professions The University of Texas Medical Branch Galveston Texas USA utmb.edu; ^4^ College of Health Sciences Sam Houston State University Conroe Texas USA shsu.edu

**Keywords:** chronic pain, clinical instructors, evidence-to-practice gap, mixed methods, pain neuroscience education, physical therapy

## Abstract

**Purpose:**

Despite advances in pain science education, evidence‐based approaches to chronic pain are not consistently translated into physical therapy practice. Physical therapist (PT) clinical instructors (CIs) occupy a unique role as both clinicians and mentors of future practitioners. This study examined PT CIs’ pain knowledge, pain‐related beliefs, confidence, and self‐reported use of pain neuroscience education (PNE) and explored perceived barriers to implementing evidence‐informed persistent pain management and preparing students to care for individuals with persistent pain.

**Methods:**

This convergent mixed‐methods study used an anonymous online survey to collect quantitative and qualitative data from 30 PT CIs. Quantitative measures assessed pain neurophysiology knowledge, pain‐related beliefs, pain care confidence, and reported use of PNE. Open‐ended responses explored challenges in chronic pain management and student mentorship. Quantitative data were summarized descriptively, qualitative responses were analyzed using reflexive thematic analysis, and findings were integrated through SCT to examine factors contributing to the evidence‐to‐practice gap.

**Results:**

Participants demonstrated high pain care confidence, moderate‐to‐high pain neurophysiology knowledge, low disability‐oriented beliefs, and moderate use of PNE. Qualitative themes identified challenges in pain communication, management of complex presentations, and patient engagement that are compounded by clinical constraints. CIs also perceived that students possessed foundational pain knowledge but struggled to apply it through communication, treatment progression, and PNE. Integrated findings revealed a mismatch between knowledge and confidence and the application of biopsychosocial pain management.

**Conclusion:**

The evidence‐to‐practice gap in chronic pain management may reflect more than inadequate pain knowledge. While CIs reported strong confidence and generally contemporary beliefs, they continued to experience difficulty with communication, adaptability, patient engagement, and implementation within constrained clinical environments. Strategies to improve pain management should therefore extend beyond knowledge acquisition and include applied communication training, context‐sensitive clinical decision‐making, and support for clinicians who model pain care for future PTs.

## 1. Introduction

Persistent pain affects approximately one in five U.S. adults, with nearly 7% experiencing high‐impact chronic pain that substantially limits daily activities [1, 2]. Persistent pain presents a complex clinical challenge for physical therapists (PTs), requiring assessment and management that extends beyond tissue pathology to incorporate biological, psychological, and social contributors to the pain experience. The theoretical foundations of this approach trace to Engel’s biopsychosocial model of illness, which called for a clinical framework incorporating biological, psychological, and social dimensions of health [3]. It was later extended to pain through Loeser’s conceptual synthesis of nociception, pain, suffering, and pain behavior [4]. The biopsychosocial model is now widely accepted as the most comprehensive perspective for understanding and treating chronic pain [5], and psychosocial factors recognize catastrophizing, fear avoidance, and social context as substantial contributors to pain‐related outcomes [3].

Contemporary pain science has advanced the understanding of persistent pain and informed recommendations for assessment and management, emphasizing individualized, biopsychosocial care and the integration of pain neuroscience education (PNE) into physical therapy practice [6, 7]. Despite these advances, implementation of contemporary pain management remains challenging. In a scoping review of 24 studies [8], it concluded that physiotherapists′ use of the biopsychosocial model for chronic pain is “far from the recommendations given in research and current guidelines” and identified barriers related to clinician knowledge, confidence, therapeutic alliance, patient expectations, role clarity, and environmental context. These findings suggest that improving implementation of evidence‐informed pain care requires more than increasing knowledge alone; understanding the factors that influence clinicians′ translation of contemporary pain science into practice is equally important [8]. These implementation challenges extend across practice contexts. In a cross‐sectional survey of 447 Spanish physiotherapists, more than half favored the biopsychosocial model of chronic pain management, yet only 9.8% reported always applying it and 40.7% frequently, with self‐reported confidence and skills among the strongest determinants of implementation. Clinicians identified inadequate psychological skills (63.6%), coordination challenges (47.6%), time constraints (43.6%), and patient misconceptions (34.2%) as key barriers [9].

Clinical instructors (CIs) occupy a unique position at the intersection of patient care and professional education. As practicing clinicians, they are responsible for implementing evidence‐informed pain management while simultaneously modeling clinical reasoning, communication, and patient‐centered care for future PTs. Role modeling is recognized as an essential competency of CIs, with students learning not only technical skills but also professional behaviors, attitudes, and approaches to clinical decision‐making through observation [10]. Furthermore, Sujee et al. [11] reported that physiotherapy students understood the principles of biopsychosocial pain management but experienced difficulty translating this knowledge into clinical practice, highlighting the importance of effective clinical supervision and role modeling during clinical education [11].

Social cognitive theory (SCT) provides the theoretical basis for understanding how this modeling influences learning. According to social learning theory, individuals learn in part by observing others and modeling their behavior within a social context, with the clinical environment representing a key determinant of what and how students learn [12]. Learning from role models is widely regarded as having the greatest impact on professional formation during clinical training [13], and medical education research demonstrates how social interactions in clinical settings give rise to learning [14]. Consequently, students may acquire not only technical competencies but also pain‐related beliefs, attitudes, and approaches to clinical decision‐making through observation of their CIs [12, 13].

Previous research has demonstrated variability in CIs’ evidence‐based practice knowledge and their modeling of evidence‐based behaviors during clinical education, while qualitative work has highlighted the importance of clinical competence, communication, confidence, and lifelong learning in effective clinical instruction [15–17]. Quantitative studies support this variability: in a survey of 376 U.S. PT CIs, 2.1% reported never integrating evidence into their clinical instruction, and only 49.5% did so frequently, with just 46% possessing the level of evidence‐based practice knowledge that CIs themselves considered more than adequate for their role [15]. Qualitative work has identified a perceived lack of role models in evidence‐based practice among both students and CIs [18]. Furthermore, CI preparation for the teaching role is itself limited as most experienced clinicians have not been formally trained in educational techniques [19]. This is significant because the learning triad of student, instructor, and clinical community illustrates how the knowledge, attributes, and skills of each party can either support or impede learning in the clinical environment [20].

Pain‐specific preparation is similarly variable across the PT workforce. Formal chronic pain training in entry‐level physical therapy curricula ranges from 5 to 115 h, and fewer than half of physical therapy students demonstrate awareness of best‐practice clinical practice guidelines for chronic pain management [21]. Dedicated pain curricula significantly improve students′ pain knowledge and psychosocial beliefs [22], whereas a strictly biomedical orientation in training can increase maladaptive fear‐avoidance beliefs and worsen the activity recommendations students provide [23]. Even among practicing PTs, confidence in managing persistent pain varies with pain science education exposure [24].

Little is known about CIs′ pain‐specific knowledge, pain‐related beliefs, confidence, use of PNE, or perceptions of barriers to implementing contemporary persistent pain management. Therefore, the purpose of this convergent mixed‐methods study was to examine PT CIs’ pain knowledge, pain‐related beliefs, confidence, and reported use of PNE while exploring their perceptions of barriers to implementing evidence‐informed persistent pain management and preparing students to care for individuals with persistent pain. By integrating quantitative and qualitative findings, this study sought to provide a more comprehensive understanding of factors that may contribute to the evidence‐to‐practice gap in persistent pain management.

## 2. Materials and Methods

The present study was informed by SCT, which proposes that behavior is shaped through the reciprocal interaction of personal factors, behaviors, and environmental influences [25]. Within the context of persistent pain management, SCT provides a useful framework for understanding how PT CIs’ knowledge, pain‐related beliefs, confidence (self‐efficacy), and clinical experiences may influence the implementation of evidence‐informed care. Behavioral capability, defined as the knowledge and skills necessary to perform a behavior, aligns with the quantitative assessment of pain knowledge, pain‐related beliefs, confidence, and self‐reported use of PNE. Reciprocal determinism recognizes that clinicians′ behaviors are influenced by interactions with environmental factors, such as patient complexity, time constraints, and workplace expectations, which were explored through the qualitative component of this study. Although CIs also serve as role models through observational learning, the primary focus of this study was to understand factors influencing their implementation of contemporary persistent pain management.

### 2.1. Study Design

This study used a convergent mixed‐methods design, in which quantitative and qualitative data were collected concurrently, analyzed separately, and integrated during interpretation to provide a more comprehensive understanding of the research problem [26]. A convergent design was selected because quantitative measures provided information regarding CIs′ pain knowledge, pain‐related beliefs, confidence, and self‐reported use of PNE, whereas qualitative responses provided insight into participants′ experiences and perceived barriers to implementing evidence‐informed persistent pain management. Integration of the quantitative and qualitative findings provided a more comprehensive understanding of PT CIs’ implementation of evidence‐informed persistent pain management than either dataset alone. Survey questions were developed through a structured, iterative process designed to maximize content relevance, clarity, and face validity.

### 2.2. Survey Development

Questions were constructed to align with the theoretical framework and key constructs of interest: pain‐related knowledge, beliefs, and confidence; approaches to treating patients with pain; and experiences as a CI working with students.

Following initial development, the questions were reviewed by two experienced physical therapy pain researchers who evaluated each item for clarity, relevance, and alignment with current pain science literature and educational constructs. Feedback from both reviewers was incorporated, and consensus was reached among the development team regarding the final wording and structure of each item prior to use in the study. This consensus process served as an initial assessment of face validity, ensuring that the questions appeared to measure the intended constructs from the perspective of content experts.

### 2.3. Participants and Recruitment

Licensed PTs serving as CIs were eligible to participate if they had supervised at least one Doctor of Physical Therapy student within the previous 10 years. Participants were recruited over a six‐month period using convenience and snowball sampling. Initial recruitment emails were distributed to clinical education coordinators and CIs affiliated with one university’s established clinical partner network through the Exxat clinical education management system. Recipients were encouraged to share the survey invitation with other eligible CIs within their organizations and professional networks. To broaden recruitment, the survey was also promoted through professional social media platforms (LinkedIn and Facebook). The study was approved by the Institutional Review Board (IRB #24‐034), and electronic informed consent was obtained from all participants before survey completion. Responses were collected anonymously. Thirty CIs completed both the quantitative and qualitative components of the survey. Most participants practiced in outpatient settings (*n* = 29), with one participant practicing in an inpatient setting. Additional participant characteristics are presented in Table 1.

**TABLE 1:** Demographics (*n* = 30).

Characteristic	*n* (%)
Female	13 (43.3%)
Highest degree earned	
Bachelor’s	1 (3.3%)
Master’s	3 (10.0%)
Doctorate	26 (86.7%)
Hours of pain science continuing education	
0 h	1 (3.3%)
1–10 h	15 (50.0%)
11–20 h	3 (10.0%)
21–30 h	3 (10.0%)
30+ hours	8 (26.7%)
Pain‐related certifications	5 (16.7%)
Board certifications	10 (33.3%)

### 2.4. Procedures

A comprehensive survey was distributed via Microsoft Forms during Fall of 2024. The survey collected demographic data, views on pain science education, the Pain Care Confidence Scale (PCCS), and the Health Care Providers’ Pain and Impairment Relationship Scale (HC‐PAIRS), and the revised Neurophysiology of Pain Questionnaire (rNPQ). Participants also reported perceived coverage of pain science in their entry‐level PT program, the amount of continued education in pain science, and the frequency of pain management interventions, including PNE, using a 5‐point Likert scale. Additionally, the survey included questions about the year graduated from PT school and average number of PT students supervised per year. Three open‐ended questions elicited qualitative data on challenges encountered in managing chronic pain, clinical barriers to pain management, and teaching strategies used during clinical education. The responses were collected in the form of typed answers in the survey.

### 2.5. Materials

Three self‐report instruments were used to assess participants’ pain‐related beliefs, knowledge, and confidence. The HC‐PAIRS measures provider attitudes and beliefs related to chronic pain and demonstrates good internal consistency and test–retest reliability [27]. The 13‐item scale asks respondents to rate their agreement with statements suggesting that pain justifies functional limitation or disability. Items are rated on a 7‐point Likert‐type scale ranging from *completely disagree* to *completely agree* and summed to produce a total score of 13–91, with higher scores reflecting stronger endorsement of the belief that chronic pain is associated with or justifies impairment and disability. The HC‐PAIRS has demonstrated acceptable internal consistency, test–retest reliability, and construct validity in health care provider samples. The rNPQ measures pain neurophysiology knowledge with good test–retest reliability [28]. The instrument includes 13 statements answered as true, false, or undecided. Responses are scored as correct or incorrect, with higher total scores indicating greater pain neurophysiology knowledge. The rNPQ results are reported as a percentage correct. The PCCS measures pain‐related confidence across assessment, treatment, and interprofessional collaboration on a 1–10 scale [29]. Participants rated five items on an 11‐point scale from 0 to 10. Item scores were averaged to yield a mean PCCS score ranging from 0 to 10, with higher scores indicating greater perceived confidence in managing persistent pain.

### 2.6. Data Analysis—Quantitative

Data were extracted from Microsoft Forms for analysis. Quantitative data were examined using descriptive statistics (means, medians, standard deviations, and ranges) for CIs’ demographics and key variables, including pain knowledge (rNPQ), biopsychosocial orientation (HC‐PAIRS), pain care confidence (PCCS), and reported use of PNE. Exploratory Spearman rank‐order correlations were conducted to examine associations among pain neurophysiology knowledge (rNPQ), pain‐related beliefs (HC‐PAIRS), perceived pain‐management confidence (PCCS), and frequency of use of PNE in practice.

### 2.7. Data Analysis—Qualitative

Qualitative data from the open‐ended responses were analyzed using reflexive thematic analysis as described by Virginia Braun and Victoria Clarke [30]. This approach was selected to identify patterns of meaning across participants′ descriptions of implementing evidence‐informed persistent pain management and supervising students in clinical practice. The analysis was conducted from a critical realist perspective, recognizing that while CIs’ pain knowledge, beliefs, confidence, and reported clinical behaviors can be examined using quantitative measures, participants′ accounts reflect individual interpretations shaped by broader clinical and organizational contexts. This perspective supported interpretation of qualitative findings related to implementation barriers, including patient complexity, time constraints, and workplace expectations, while acknowledging that these contextual influences interact with clinicians′ knowledge and confidence. Integration of the quantitative and qualitative findings provided complementary perspectives on CIs′ implementation of evidence‐informed persistent pain management, allowing standardized measures to be interpreted alongside participants′ descriptions of implementation challenges. This approach provided a more comprehensive understanding of factors that may contribute to the evidence‐to‐practice gap in persistent pain management without implying causal relationships.

Analysis followed a multiphase process. One investigator first familiarized themselves with the data by reviewing all responses in full. Responses were then divided by meaning and coded, with codes representing patterns of shared meaning. *Inductive* coding was conducted manually by organizing data excerpts and grouping them based on conceptual similarity. Reflexive analytic memos were maintained throughout to document decisions and insights.

Codes were then actively organized into broader candidate themes through an iterative process of comparing, refining, and collapsing related concepts. Following Braun and Clarke’s guidance, theme development prioritized conceptual relevance to the research questions over numerical prevalence [16]. Themes were developed based on patterns of meaning rather than frequency counts, with attention to capturing the complexity and nuance of participants’ experiences. A third‐party reviewer conducted a peer debriefing, independent of both data collection and coding, examining code groupings and thematic interpretations to challenge assumptions and strengthen credibility. Themes were subsequently refined through team discussion. Consistent with reflexive thematic analysis, the goal was not to achieve data saturation but rather to develop an interpretive understanding of patterned meaning across participants’ responses.

A second investigator independently reviewed the dataset and developed preliminary themes. The research team then engaged in collaborative discussions to compare interpretations, examine points of convergence and divergence, and refine theme definitions. This process emphasized reflexivity and interpretive depth rather than inter‐rater reliability.

Themes were reviewed and refined to ensure internal coherence and distinctiveness, resulting in a final set of overarching themes.

### 2.8. Data Analysis—Mixed Methods Integration

The analysts were PT faculty with experience in persistent pain management, pain science education, and clinical instruction. While this background provided valuable clinical and educational insight, it also introduced potential assumptions regarding the implementation of evidence‐informed persistent pain management and knowledge translation. To enhance reflexivity, the research team engaged in reflexive memoing throughout the analytic process and used peer debriefing to challenge interpretations and consider alternative perspectives. Integration of the qualitative findings with the quantitative results further supported critical reflection within the convergent mixed‐methods design.

SCT guided interpretation of the integrated quantitative and qualitative findings by providing a framework through which the results were understood in relation to self‐efficacy, behavioral capability, observational learning, and reciprocal determinism. Themes related to communication challenges, inconsistent implementation of PNE, and environmental barriers were interpreted in relation to clinicians′ knowledge, pain‐related beliefs, confidence, and the clinical contexts in which evidence‐informed persistent pain management was implemented. This framework provided a conceptual lens for interpreting the relationships among clinicians′ knowledge, beliefs, confidence, and reported implementation experiences within the broader clinical environment.

## 3. Results

### 3.1. Quantitative Results

Participants (*n* = 30) were predominantly doctoral‐trained PTs, with 50% practicing in rural or underserved communities. The median year of graduation from physical therapy school was 2014 (range: 1988–2021). Notably, 11 of the 30 participants (36.7%) graduated between 2018 and 2021, following the International Association for the Study of Pain (IASP) revised pain curriculum guidelines that were published in 2018, while the remaining 19 participants (63.3%) graduated prior to these updated recommendations. Regarding clinical teaching load, the majority of participants (*n* = 28, 93.3%) reported supervising an average of 1–4 physical therapy students per year, while 2 participants (6.7%) reported supervising an average of 5–8 students per year. Nearly half reported between 1 and 10 h of continuing education in pain science, and approximately one‐third held board certifications, while 16.7% reported pain‐specific certifications (Table 1). On standardized measures, respondents demonstrated high confidence in managing pain, moderate‐to‐high knowledge of pain neurophysiology, and generally low agreement with beliefs that chronic pain justifies disability, with moderate application of PNE in practice (Table 2). Frequency of PNE use was positively associated with PCCS scores, *r*_*s*_(28) = 0.37, *p* = 0.045, indicating that more frequent reported PNE use was associated with greater perceived confidence in pain management. No statistically significant associations were observed among the remaining variables.

**TABLE 2:** Summary of pain‐related measures.

Measure	Description	Mean (SD)	Range
Pain Care Confidence Scale (PCCS) Likert scale	Confidence in managing patients with pain. Item responses were averaged to create a mean PCCS score ranging from 0 to 10. Higher scores indicate greater perceived confidence; 0 indicates no confidence, and 10 indicates complete confidence.	7.20 (1.5)	3.4–10
Revised Neurophysiology of Pain Questionnaire (rNPQ)	Knowledge of pain science. Item responses. Responses were calculated as percentage correct.	75.8% (13%)	33%–92%
Health Care Providers’ Pain and Impairment Relationship Scale (HC‐PAIRS)	Beliefs about pain‐related disability. Used the 13‐item with scores ranging from 13 to 91; higher scores reflect a stronger belief that chronic pain is directly associated with, and justifies, functional impairment and disability; lower scores reflect less endorsement of this belief.	35.6 (7.65)	21–52
Likert scale (1 = *never* to 5 = *always*)	Use of pain neuroscience education (PNE) in practice	3.57 (1.36)	1–5

*Note:* HC‐PAIRS, Health Care Providers’ Pain and Impairment Relationship Scale.

Abbreviations: PCCS, Pain Care Confidence Scale; PNE, pain neuroscience education; rNPQ, revised Neurophysiology of Pain Questionnaire.

### 3.2. Qualitative Results

Fifteen codes were organized into themes, which were collapsed into the final three per question based on theoretical relevance to SCT constructs. Responses to the third open‐ended question did not yield sufficient depth or patterned meaning relevant to the study aims and were therefore excluded from thematic development. Thematic quotes are displayed in Table 3.

**TABLE 3:** Qualitative themes and illustrative quotations.

Survey question	Theme	Illustrative participant quotations
Q1: Student preparation for persistent pain management	Translating pain knowledge into clinical practice	“They can assess it and identify when a patient has chronic pain but often lack ability to effectively communicate with these patients and modify interventions.” • “They can understand the pain and where it is coming from, but understanding why the pain is persisting they struggle with.” • “They are unfamiliar with any specific treatment approaches such as PNE.”
	Communication and confidence barriers	“Students are fearful of pain and worried they will make people have more pain with interventions.” • “The biggest weakness that I see…is fear of pushing these patients appropriately and the confidence in explaining pain neuroscience to patients.” • “Weaknesses are lack of full understanding, limited confidence in treating those with chronic pain, and at times lack of confidence/experience in modifying treatments to meet the level of the patient.”
	Educational priorities for implementing evidence‐informed persistent pain management	“Students need taught that there is no ‘black and white’ situation, that every situation is different and you must get the patient to ‘buy in’ to therapy.” • “I would recommend more pain neuroscience education to DPT students. Educate them more on the theories behind chronic pain and the best practices to manage it.” • “Students learn so much in terms of skills and techniques, but application to patient care would be helpful.”

Q2: Challenges managing patients with persistent pain	Communicating contemporary pain concepts with patients	“Getting them to buy into the education aspect of pain and understanding it isn’t just tissue damage.” • “I feel that I do well trying to explain chronic pain in regards to the pathophysiology, however it often seems like it is abstract to patients…So, overall I feel it is difficult to describe in a concrete, patient centered manner.” • “The biggest challenge…is having them understand why they are having pain as well as ways to help treat their pain.”
	Clinical and environmental barriers to implementing persistent pain management	“The system in which I operate. If you are a busy practice that does not support 1 on 1 time, treating these patients can be difficult.” • “Time availability within the clinic with a standard caseload of an outpatient environment.” • “I feel like I have to change the treatment each visit based on how my chronic pain patients are presenting.”
	Patient engagement and readiness for behavior change	“Patient buy‐in is the hardest part for me.” • “Their willingness sometimes to try new things.” • “Fear‐Avoidance Beliefs, patients feeling that their pain will never get better, lack of HEP compliance…many have made up their mind that nothing will help them.”

Question 1**:** What do you perceive are the strengths and weaknesses of DPT students on clinical rotations in managing patients with persistent/chronic pain? Please describe what you would recommend to DPT programs for better preparing students to manage patients with persistent/chronic pain on clinical rotations.

Theme 1: Translating pain knowledge into clinical practice. CIs consistently perceived that students entered clinical rotations with foundational knowledge of pain mechanisms and basic clinical skills but often struggled to translate this knowledge into the management of individuals with persistent pain. Participants noted that students could identify pain conditions and perform interventions but described challenges applying PNE, communicating pain concepts, and progressing treatment for patients with more complex pain presentations. One CI described students as having a “good understanding of different types of pain,” whereas another reported that students were “unfamiliar with PNE” and had “difficulty with where to start treatment.” Several participants also perceived that students expected relatively rapid improvement and were “quick to decide a patient will not benefit from therapy when they do not progress quickly,” reflecting difficulty applying contemporary pain principles in complex clinical situations.

Theme 2: Communication and confidence barriers. Participants described communication and clinical confidence as challenges for students managing individuals with persistent pain. CIs perceived that students often struggled with the emotional and interpersonal demands of persistent pain management, particularly when patients demonstrated slow or inconsistent progress. Responses reflected uncertainty when modifying treatment plans, communicating with patients about persistent pain, and maintaining confidence in clinical decision‐making. Several participants described students as “fearful of making pain worse” or “taking on the patient’s pain,” suggesting that concerns about causing harm and emotional investment in patient outcomes may influence confidence and communication during clinical encounters.

Theme 3: Educational priorities for implementing evidence‐informed persistent pain management. Participants identified several educational priorities that they believed would better prepare students to implement evidence‐informed management of persistent pain in clinical practice. CIs emphasized the need for greater exposure to case‐based learning, biopsychosocial approaches to pain assessment and management, and communication strategies that reflect the complexity of persistent pain. One participant stated that students “need to be taught that there is no black and white situation,” while another emphasized the importance of “teaching the psychology of chronic pain.” Participants also highlighted the need for learning experiences that support clinical reasoning, treatment progression, and the integration of PNE into patient care.

Question 2: Please describe what your biggest challenges have been in the clinical management of patients with persistent/chronic pain.

Theme 1: Explaining pain in a patient‐centered way. Participants consistently described patient communication as one of the most challenging aspects of implementing evidence‐informed persistent pain management. CIs reported difficulty explaining contemporary pain concepts in ways that patients could understand, particularly when symptoms persisted despite the absence of clear tissue pathology or when patient expectations differed from contemporary pain science. Responses reflected challenges communicating complex and often abstract pain mechanisms while addressing patients′ questions and concerns. One CI described “struggling to communicate that it is more than tissue damage,” while another noted difficulty “fielding the ‘why’ questions” from patients.

Theme 2: Medical management and environmental barriers. Participants described the implementation of evidence‐informed persistent pain management as challenging because of both the complexity of persistent pain presentations and the realities of the clinical environment. CIs emphasized the need to continually adapt treatment approaches to patients with variable symptom presentations while coordinating care within interdisciplinary healthcare systems. Participants also identified environmental constraints, including limited treatment time, productivity expectations, and large caseloads, as factors that reduced opportunities for individualized patient education and management.

Theme 3: Engagement and motivation barriers. Participants described patient engagement as an ongoing challenge when implementing evidence‐informed persistent pain management. CIs frequently identified fear‐avoidance behaviors, inconsistent participation in home programs, and reluctance to adopt new management strategies as barriers to patient progress. Several participants described patients as “going through the motions” or demonstrating “fear avoidance,” highlighting the challenges of supporting behavior change and sustained engagement in rehabilitation among individuals with persistent pain.

Question 3: Please describe what teaching strategies you have utilized with your DPT students to facilitate learning during clinical education?

Although participants reported instructional approaches such as verbal discussion, demonstration, feedback during patient care, and case‐based learning, responses were generally brief and descriptive, and represented a broad range of teaching strategies without sufficient patterned meaning specifically related to persistent pain to warrant thematic development**.** Therefore, these responses were not incorporated into the integrated thematic findings.

### 3.3. Mixed Methods Integration

Integration of the quantitative and qualitative findings demonstrated convergence between CIs’ self‐reported pain knowledge, beliefs, confidence, and their descriptions of implementing evidence‐informed persistent pain management. Quantitatively, participants demonstrated moderate‐to‐high pain knowledge, generally low disability‐oriented beliefs, high confidence, and moderate self‐reported use of PNE. Qualitative findings complemented these results by illustrating that, despite favorable knowledge and belief profiles, CIs continued to describe challenges communicating contemporary pain concepts, managing complex clinical presentations, navigating environmental constraints, and supporting patient engagement.

Responses regarding student learning similarly complemented the quantitative findings. Although CIs perceived that students entered clinical education with foundational pain knowledge, they frequently described difficulty translating that knowledge into the application of PNE, clinical reasoning, and treatment progression for individuals with persistent pain. Together, the quantitative and qualitative findings suggest that implementation of evidence‐informed persistent pain management may require more than adequate knowledge and confidence alone, with contextual and communication‐related factors emerging as important considerations.

The integrated findings also highlighted areas where the qualitative data expanded the interpretation of the quantitative results. Although CIs reported favorable pain knowledge, beliefs, and confidence, their qualitative descriptions demonstrated that these attributes could coexist with persistent implementation challenges in complex clinical environments. Rather than contradicting the quantitative findings, the qualitative data provided important context by illustrating how communication demands, environmental constraints, and patient‐related factors may influence the implementation of evidence‐informed persistent pain management.

Exploratory comparison of quantitative profiles with qualitative themes suggested that the nature of reported challenges was not consistently associated with pain knowledge alone. CIs who described difficulty communicating pain concepts or managing complex clinical presentations demonstrated similar or somewhat higher pain knowledge than the overall sample, while reporting lower confidence. In contrast, CIs who emphasized patient engagement and motivation barriers demonstrated similar pain knowledge but lower self‐reported PNE use and confidence. Given the small sample and overlapping theme membership, these observations were interpreted descriptively rather than as subgroup differences.

## 4. Discussion

This convergent mixed‐methods study examined PT CIs′ pain knowledge, pain‐related beliefs, confidence, and self‐reported use of PNE while exploring their experiences implementing evidence‐informed persistent pain management. Quantitatively, participants demonstrated moderate‐to‐high pain knowledge, generally supportive pain beliefs, high confidence, and moderate self‐reported use of PNE. Qualitative accounts added context to the quantitative results by showing how clinicians encountered practical barriers when translating pain knowledge into day‐to‐day practice. Participants described difficulties tailoring contemporary pain explanations, responding to complex presentations, working within environmental and organizational constraints, and sustaining patient engagement. These experiences suggest that implementation is shaped not only by clinicians’ knowledge and confidence but also by communication skills, clinical context, and available support.

CIs also perceived that students generally entered clinical education with foundational pain knowledge but experienced difficulty translating that knowledge into patient‐centered management of persistent pain. Participants frequently described challenges related to applying PNE, adapting treatment progression, and communicating contemporary pain concepts in complex clinical situations. These perceptions complemented the quantitative findings by suggesting that implementation challenges may extend across both experienced clinicians and learners, although the present study did not directly evaluate student knowledge or performance.

These findings are consistent with a growing body of implementation research suggesting that contemporary pain science has not been uniformly translated into clinical practice. Van Dijk et al. identified clinician confidence, therapeutic alliance, patient expectations, environmental context, and role clarity as important barriers to implementing biopsychosocial pain management [8]. Similarly, participants in the present study described communication challenges, complex patient presentations, environmental constraints, and patient engagement as persistent obstacles despite generally favorable knowledge and belief profiles.

Participants in the present study demonstrated relatively low disability‐oriented pain beliefs, with a mean HC‐PAIRS score of 35.6. This score was lower than the mean score of 46.30 reported for PTs in the 2024 scoping review by Müller et al., which included 14 studies and 5,055 PTs [31]. Because higher HC‐PAIRS scores indicate greater endorsement of the belief that chronic pain is closely linked to, or justifies, functional impairment and disability, the lower score in the present sample suggests that CIs may hold comparatively more contemporary and less disability‐oriented beliefs about persistent pain. This pattern may be particularly relevant given CIs’ roles in modeling clinical reasoning and pain‐related communication for students. However, this comparison should be interpreted cautiously because the studies included in the review likely differed from the present sample in country, clinical setting, years of experience, professional training, exposure to contemporary pain education, and HC‐PAIRS administration and scoring procedures. Therefore, the observed difference should be viewed as contextual rather than evidence that CIs hold definitively more evidence‐informed pain beliefs.

Participants’ mean rNPQ score was 75.8%, indicating that, on average, respondents answered approximately three‐quarters of pain neurophysiology knowledge items correctly. This score was lower than the mean of 82.5% reported by Keter et al. in their 2025 sample of 134 PTs, suggesting comparatively lower pain neurophysiology knowledge in the present sample [32]. In contrast, the present mean was higher than the 61.4% reported by Ehrström et al. in 2024 among 202 PTs [33]. Taken together, these findings place the present sample’s rNPQ performance between those of the two comparator PTs samples.

The variability across studies may reflect differences in prior education in pain science, continuing professional development, professional role, clinical experience, participant selection, and use of translated or culturally adapted rNPQ versions. Accordingly, the present score should not be interpreted as an absolute indicator of adequate or inadequate knowledge. Rather, it suggests that CIs had a moderate‐to‐high level of conceptual pain knowledge relative to published PT samples, while still leaving potential room for additional education or support in translating knowledge into clinical communication and management of complex persistent‐pain presentations.

The qualitative findings extended the quantitative results by providing contextual insight into the challenges CIs encounter when implementing evidence‐informed persistent pain management. While quantitative measures suggested generally favorable pain knowledge, beliefs, and confidence, participants′ narratives illustrated that translating these attributes into clinical practice was influenced by communication challenges, environmental constraints, and patient‐related factors. These complementary findings illustrate the value of a convergent mixed‐methods approach for understanding implementation beyond what standardized measures alone can capture.

SCT provided a useful framework for interpreting the integrated findings. CIs′ pain knowledge, beliefs, and confidence represent personal factors that may influence implementation behaviors, while the qualitative findings highlighted environmental influences, including patient complexity, workplace expectations, and time constraints. Viewed together, these findings support SCT’s proposition that clinical behavior emerges through interactions among individual capabilities, environmental context, and behavioral experiences rather than through knowledge alone.

## 5. Educational and Clinical Implications

The findings of this study suggest that supporting the implementation of evidence‐informed persistent pain management may require more than expanding clinicians′ pain‐related knowledge. Although CIs demonstrated favorable pain knowledge, beliefs, and confidence, the qualitative findings highlighted ongoing challenges related to communication, management of complex clinical presentations, and consistent application of PNE. Because CIs also serve as role models for future PTs, strengthening these implementation skills has the potential to influence both contemporary clinical practice and student learning. Participants′ perceptions that students experienced similar challenges translating foundational knowledge into clinical practice further suggest that educational experiences should emphasize communication, clinical reasoning, and application of evidence‐informed persistent pain management within authentic clinical contexts. These findings are consistent with implementation research suggesting that improving uptake of contemporary pain management requires attention not only to clinician knowledge but also to communication, contextual barriers, and opportunities for supported application in clinical practice [8].

### 5.1. Limitations

Several limitations should be considered when interpreting these findings. First, participants were recruited using convenience and snowball sampling through one university’s clinical partner network and professional social media platforms. Consequently, the sample may overrepresent CIs with greater interest in persistent pain management or professional development, limiting the generalizability of the findings. The CIs who participated had a range of specific pain training and do not represent a sample of CIs with advanced pain training. The relatively small sample size also precluded meaningful subgroup analyses and supports the interpretation of the quantitative findings as exploratory rather than definitive.

Second, although the survey was developed through an iterative process informed by clinical and pain science expertise, it did not undergo formal psychometric validation or pilot testing. Furthermore, the open‐ended question regarding teaching strategies yielded relatively brief, general descriptions of instructional approaches rather than detailed accounts of persistent pain management, limiting the depth of qualitative analysis for that aspect of the study. Future research using interviews or focus groups may provide richer insight into how CIs teach and model evidence‐informed persistent pain management during clinical education.

## 6. Conclusion

This convergent mixed‐methods study provides insight into PT CIs′ implementation of evidence‐informed persistent pain management. Although participants demonstrated moderate‐to‐high pain knowledge, generally supportive pain beliefs, high confidence, and moderate self‐reported use of PNE, the qualitative findings highlighted persistent challenges among clinicians and students related to communication, complex clinical presentations, environmental constraints, and patient engagement. Together, these findings suggest that successful implementation of evidence‐informed persistent pain management may require more than knowledge and confidence alone.

Because CIs serve as both practicing clinicians and role models for future PTs, supporting the translation of contemporary pain science into clinical practice may benefit both patient care and clinical education. Educational and professional development initiatives that emphasize communication, clinical reasoning, and application of evidence‐informed persistent pain management may further support implementation in complex clinical environments.

## Funding

No funding was received for this manuscript.

## Ethics Statement

Proposal number 24‐034 approved as exempt by the University of Charleston IRB.

## Conflicts of Interest

The authors declare no conflicts of interest.

## Data Availability

The data that support the findings of this study are openly available in Dataverse at https://doi.org/10.7910/DVN/7GGISW.
